# A Micro Insight of Water Permeation in Polyurethane: Navigating for Water Transport

**DOI:** 10.3390/polym17020129

**Published:** 2025-01-07

**Authors:** Kai Chen, Zhenyuan Hang, Yongshen Wu, Chao Zhang, Yingfeng Wu

**Affiliations:** 1 College of Road and Bridge, Zhejiang Institute of Communications, Hanghzou 311112, China; zjchenkai@hotmail.com (K.C.); wuyingf_hz@hotmail.com (Y.W.); 2 School of Water Conservancy and Transportation/Yellow River Laboratory/Underground Engineering Institute, Zhengzhou University, Zhengzhou 450001, China; chao.zhang.zzu@outlook.com

**Keywords:** polymer grouting material, water, permeation, nanochannels, molecular dynamics

## Abstract

Polyurethane (PU) grouting materials are widely used in underground engineering rehabilitation, particularly in reinforcement and waterproofing engineering in deep-water environments. The long-term effect of complex underground environments can lead to nanochannel formation within PU, weakening its repair remediation effect. However, the permeation behavior and microscopic mechanisms of water molecules within PU nanochannels remain unclear. In this paper, a model combining PU nanochannels and water molecules was constructed, and the molecular dynamics simulations method was used to study the effects of water pressure and channel width on permeation behavior and microstructural changes. The results reveal a multi-stage, layered permeation process, with significant acceleration observed at water pressures above 3.08 MPa. Initially, water molecules accelerate but are then blocked by the energy barrier of PU nanochannels. After about 20 ps, water molecules overcome the potential barrier and enter the nanochannel, displaying a secondary acceleration effect, with the maximum permeation depth rises from 1.8 nm to 11.8 nm. As the channel width increases, the maximum permeation depth increases from 7.5 nm to 11.6 nm, with the rate of increase diminishing at larger widths. Moreover, higher water pressure and wider channels enhance the stratification effect. After permeation, a hydrophobic layer of approximately 0.5 nm thickness forms near the channel wall, with a density lower than that of the external water. The middle layer shows a density slightly higher than the external water, and the formation of hydrogen bonds between water molecules increases toward the channel center.

## 1. Introduction

Polyurethane (PU) grouting materials have been widely used in underground engineering rehabilitation due to their excellent mechanical properties [1], lightweight nature [2], and high chemical stability [3], particularly in reinforcement and waterproofing applications for tunnels, reservoirs, subways, and dams [4,5]. However, the underground service environment is complex, with adverse factors such as high water pressure, temperature fluctuations, and chemical erosion [6,7,8,9,10]. Furthermore, process defects, which are difficult to avoid during grouting, can exacerbate these issues. PU may deteriorate over long-term use. When PU is damaged, interconnected nanochannels may form within the material, significantly impacting its waterproofing performance and repair effectiveness and potentially leading to structural failure. Therefore, further research is required to understand the permeation behavior and molecular mechanisms of water in PU nanochannels, particularly under high water pressure conditions, where the permeation characteristics and flow patterns of water molecules are still unclear.

In recent years, the impermeability of polymers, particularly PU, has garnered significant attention. It has been found that, due to the hydrophobicity of PU, water reaches saturation at lower concentrations (2.48–2.67 wt%) in PU [11]. Once water enters the PU network, it causes plasticization of the material, and the accumulation of plasticization leads to a reduction in both the mechanical properties [11,12,13,14] and the glass transition temperature [10,11,15,16] of the PU. Water absorption in foamed PU is influenced by several factors, including environmental temperature [2,12,16,17,18], material composition [2], density [12], cell distribution [19], cell wall strength [19], water quality, and curing temperature [20]. In addition, the integrity of foamed PU is a key factor influencing its impermeability. Defects, such as cracks, openings, and wrinkles in the cell walls, can accelerate the penetration of water molecules [20,21,22,23]. For a long time, classical Fick’s law has been used to study the diffusion behavior of water molecules in polymers [15]. Some studies, however, have shown that water molecules undergo multi-stage diffusion in closed-cell PU, deviating from classical Fick’s law. To address this deviation, a multi-stage diffusion model with time-dependent diffusion rates was proposed to more accurately describe the water molecule diffusion behavior in closed-cell PU [24]. Additionally, the permeability and diffusion properties of water molecules are influenced by their structural arrangement within the polymer network. There are two main types of water molecules in polymer networks: free water and bound water [15,25]. Free water does not form hydrogen bonds (H-bonds) with the polymer network and is distributed within the free volume of the network. Bound water molecules form a large number of H-bonds with the polymer network due to their strong interactions. During the initial stage of penetration, water molecules accumulate on the cell surface and form H-bonds with polar functional groups (-NH-, -O-, -C=O, etc.) [12]. Then, more water molecules penetrate the interior of PU, accumulating in the free volume and forming clusters through intermolecular interactions. At this stage, more H-bonds form both between water molecules and between water molecules and the polymer, disrupting the initial H-bonds and van der Waals interactions between polymer chains. This enhances the mobility of polymer segments, further accelerating the permeation of water molecules [15,25]. Under the combined effects of high water pressure, initial foaming defects, external loads, and other factors, interconnected nanochannels may form within the foamed PU. However, the permeation behavior of water molecules in PU nanochannels under high water pressure remains unclear. In addition, due to limitations in instrument accuracy and scale, macroscopic experiments and finite element simulations are difficult to quantitatively characterize the microstructure and motion of water molecules within polymer networks. Therefore, molecular-scale simulations are essential to investigate the permeation characteristics and molecular mechanisms of water molecules in PU nanochannels.

Molecular dynamics (MD) simulations replicate the dynamic behavior of materials at the atomic scale by solving the equations of atomic motion, effectively overcoming the limitations of macroscopic experiments. In recent years, MD simulations have been extensively used to investigate the microscopic properties of both polymers [26,27,28,29,30] and water [31,32], with a particular focus on the microstructure and diffusion behavior of water molecules within polymer networks [33,34,35,36]. It has been found that the chemical structure of polymers significantly affects the absorption and diffusion behavior of water molecules, as variations in the chemical structure lead to notable differences in the polarity and free volume fraction of the polymer network [37]. Highly polar molecular structures tend to adsorb water molecules, thereby reducing their diffusion capacity [38]. A decrease in the free volume fraction limits the available space of water molecules and reduces their diffusion capacity, but the distribution of free volume has little effect on their diffusion behavior [39]. After crosslinking, the free volume of polymers decreases, leading to a significant reduction in the permeability of water molecules within the crosslinked network [40,41]. When water molecules penetrate the interior of PU, they form H-bonds with certain PU segments, making their diffusion behavior within the material dependent on concentration. As the concentration of water molecules increases, their diffusion capacity decreases and then gradually stabilizes [42]. At higher concentrations, water molecules tend to form clusters within PU rather than diffuse as individual molecules. H-bonds within water molecule clusters reduce the mobility of the water molecules [24]. Additionally, studies have investigated the permeation damage caused by water molecules to PU under high water pressure, finding that PU exhibits strong resistance to this damage. However, under sustained high water pressure, the accumulation of molecular chain slippage causes the free volume within the polymer network to gradually evolve into larger void defects, eventually leading to permeation failure [43]. It is believed that, after long-term use in complex service environments, nanochannels of varying sizes will form within the PU material. Research on other materials has shown that the presence of nanochannels significantly influences the structure and behavior of water molecules motion [44,45,46]. However, most existing research has focused on the microstructure and motion behavior of water molecules within polymer networks, while the microstructure and transport properties of water molecules within PU nanochannels remain unclear.

This study offers a quantitative analysis of water permeation dynamics and the associated microstructural transformations, which can provide valuable insights into how water pressure and channel width influence permeation velocity, depth, and stratification, offering valuable guidance for optimizing PU grouting applications in adverse environments. MD simulations were employed to simulate the permeation process of water molecules through PU nanochannels under high water pressure. During the permeation process, the permeation flux, depth, rate, density variation, and velocity change of water molecules were calculated under different water pressures and channel widths. The effects of water pressure and channel width on the permeation behavior of water in PU nanochannels were analyzed quantitatively. After the model reached a stable permeation state, it was further relaxed. Then, the mean square displacement (MSD), velocity change, density variation, dipole angle distribution, and H-bond distribution of water molecules inside and outside the nanochannel were calculated, enabling a quantitative analysis of the effects of PU nanochannels on the motion behavior and microstructure of water molecules.

## 2. Simulation Method

### 2.1. Force Field and Simulation Details

In this work, the optimized potentials for liquid simulations all-atom (OPLSAA) force field, which is widely used for simulating the microstructure and micromechanical properties of PU, was employed to describe the atomic interactions within PU. The total potential energy of the system is expressed by Equation (1):

(1) $$E_{\mathrm{total}}=E_{bond}+E_{angle}+E_{dihedrals}+E_{nonbonded}$$

where $E_{bond}$, $E_{angle}$, $E_{dihedrals}$, and $E_{nonbonded}$ are the bond energy, angle energy, dihedral angle energy, and non-bonded interaction energy, respectively. The individual energy terms are given as

(2) $$E_{bond}=\sum_{i}k_{b}{\left(r_{i}-r_{0}\right)}^{2}$$

(3) $$E_{angle}=\sum_{i}k_{\theta }{\left(\theta_{i}-\theta_{0}\right)}^{2}$$

(4) $$E_{dihedrals}=\sum_{i}\left[\begin{array}{l} \frac{1}{2}V_{1}\left(1+\cos\varphi_{i}\right)+\frac{1}{2}V_{2}\left(1-\cos2\varphi_{i}\right)\\ +\frac{1}{2}V_{3}\left(1+\cos3\varphi_{i}\right)+\frac{1}{2}V_{4}\left(1-\cos4\varphi_{i}\right) \end{array}\right]$$

(5) $$E_{nonbonded}=\sum_{i}\sum_{j>i}\left\{\frac{Cq_{i}q_{j}}{r_{ij}}+4\varepsilon_{ij}\left[{\left(\frac{\sigma_{ij}}{r_{ij}}\right)}^{12}-{\left(\frac{\sigma_{ij}}{r_{ij}}\right)}^{6}\right]\right\},r\leq r_{c}$$

where *k_b_* and *k_θ_* represent the stiffness of the bond tension and bond angle bending, respectively. *r*_0_ and *θ*_0_ are the equilibrium bond length and bond angle, respectively. $\varphi_{i}$ is the dihedral angle, and *V*_1_, *V*_2_, *V*_3_, and *V*_4_ are the coefficients used to describe the torsion of the dihedral angle $\varphi_{i}$. *σ_ij_* and *ε_ij_* in the Lennard-Jones potential are the equilibrium distance and the potential well depth between the two particles, respectively. *r_ij_* is the distance of the two particles, and *r_c_* is the cutoff distance of the van der Waals interaction. *C* is an energy-conversion constant, and *q_i_* and *q_j_* are the atomic charge. All the force field parameters are sourced from the literatures [47,48,49].

The SPC/E model was used to simulate the interactions between water molecules, effectively capturing the physical and thermodynamic properties of water molecules through a simplified three-point charge model. The Lorentz–Berthelot mixing rule was applied to calculate the interactions between PU and water molecules. In this study, all calculations were performed using LAMMPS software (Version 2 Aug 2023, Sandia National Laboratories, Albuquerque, NM, USA). The frog leaping method was used to calculate integrals with a time step of 0.5 fs. The atomic charge was calculated using the *qeq* method, and the long-range Coulomb interactions in the system were calculated using the Particle–Particle Mesh (PPPM) algorithm to improve the computational efficiency of charge interactions. During the simulation, the Nose–Hoover thermostat and barostat were employed to control temperature and pressure [50]. The cutoff radius for all non-bonded interactions was set to 1.2 nm. The model visualization was performed using Visual Molecular Dynamics (VMD, Version 1.9.4) software.

### 2.2. Model Construction

**Figure 1:**
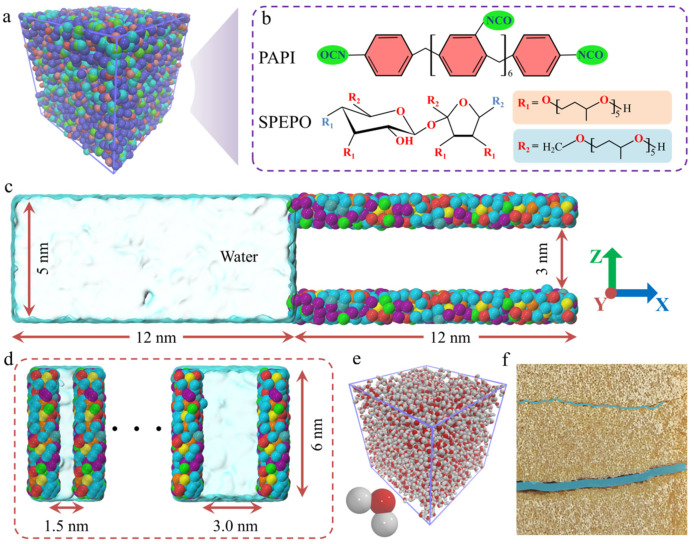
**PU nanochannel and water molecule model.** (**a**) PU molecular model in a stable state after relaxation. (**b**) Chemical structure of PAPI and SPEPO components. (**c**) Front view of the combined PU nanochannel and water molecule model. (**d**) Vertical view of the combined PU nanochannel and water molecule model. (**e**) Water molecule model. (**f**) Schematic representation of water molecules penetrating PU.

In this study, the model consists of PU nanochannels and water molecules. The PU molecular model is shown in Figure 1a, containing 10 PU chains with a total of 20,790 atoms. The PU chain was constructed using MS software (Version 2023) and consists of PAPI and SPEPO. The NCO groups at both ends of the PAPI and the OH groups at both ends of the SPEPO (the blue R1 or R2) are connected end to end. The model was then equilibrated under the NVT (i.e., constant number of atoms: N; constant volume, V; and constant temperature, T) ensemble, allowing the molecular chains to reach thermal equilibrium while keeping the volume constant. Subsequently, the system was further equilibrated under the NPT (i.e., constant number of atoms: N; constant pressure, P; and constant temperature, T) ensemble to achieve equilibrium in both density and energy. Once the model reached a stable state, two virtual planes perpendicular to the *Z*-axis were introduced using the “fix indent/plane” command. The PU model was reshaped into a flat plate under the influence of the two virtual planes. Then, two identical PU plates were positioned at different heights along the *Z*-axis to form PU nanochannels with varying widths, as shown in Figure 1c. In this study, the widths of the PU nanochannels are 1.5, 2.0, 2.5, and 3.0 nm. Packmol software (Version 20.14.4) was used to construct the water molecule model, ensuring that its height along the *Z*-axis was consistent with the height of the PU nanochannel. All water molecule models have the same length along the *X*-axis and *Y*-axis. The combination of the PU nanochannel model and the water molecule model was also carried out using Packmol software. The combined model was then relaxed for 2 ns in the NVT ensemble.

### 2.3. Penetration Simulation

Water molecules with a thickness of 2 nm on the left side of the model were treated as rigid bodies. A constant force was then applied to the remaining rigid water molecules using the “fix addforce” command [43,51]. By adjusting the magnitude of the applied force, various water pressures were simulated on the water molecules. The water pressure can be calculated using Equation (6) as follows:

(6) $$P=\frac{F\cdot N_{water}}{A}$$

where *P* represents the water pressure, *F* represents the force applied to each atom, *N_water_* represents the number of water molecules in the rigid body region, and *A* represents the cross-sectional area of the model in the direction of permeation. In this study, water pressures of 1.03, 2.05, 3.08, 4.10, 5.12, and 6.16 were applied to investigate the effect of pressure on the permeability characteristics of water molecules. Additionally, the number of water molecules in the rigid body region varies among models with different widths. To study the effect of nanochannel width on water molecule permeability, the magnitude of the force applied to the rigid water molecules was adjusted, ensuring that models with varying widths are simulated under the same pressure. In this study, the simulation time for water molecule permeation in all models was set to 100 ps.

## 3. Results and Discussion

### 3.1. The Effect of Water Pressure

**Figure 2:**
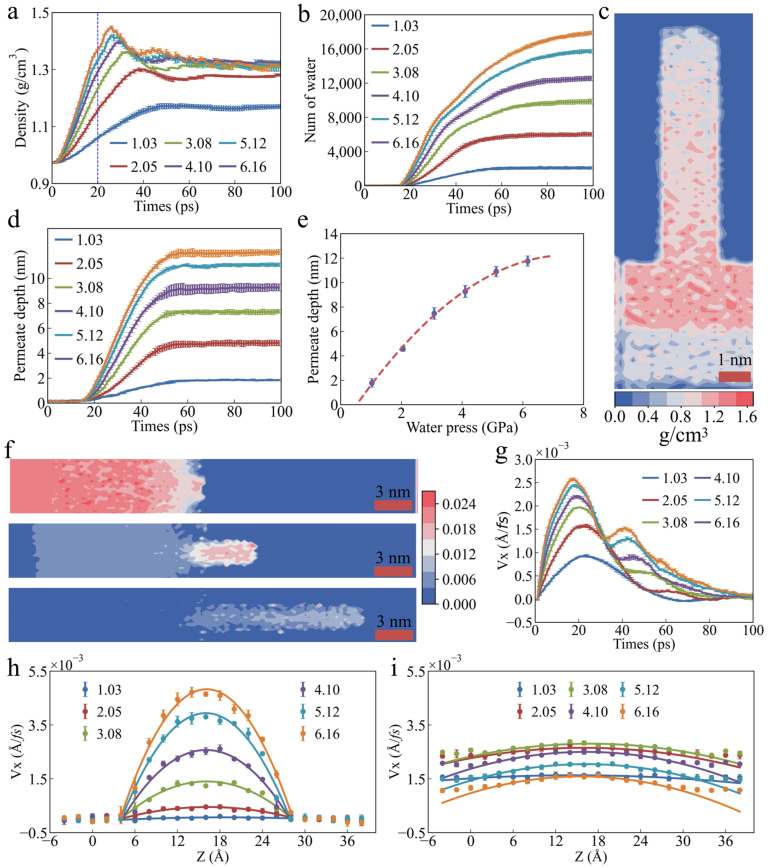
**Effect of water pressure on the permeability characteristics of water molecules.** (**a**) Average water density as a function of permeation time. (**b**) Number of water molecules permeating PU nanochannels as a function of permeation time. (**c**) Two-dimensional density distribution of water molecule at stable permeation. (**d**) Permeation depth as a function of permeation time. (**e**) Maximum permeation depth as a function of water pressure. (**f**) Two-dimensional velocity distribution of water molecules during permeation at a water pressure of 6.06 MPa. (**g**) Average velocity of water molecules as a function of permeation time. Distribution of permeation velocity of water molecules along the *Z*-Axis (**h**) inside and (**i**) outside nanochannels. The unit of atomic velocity is Å/*f*s.

**Figure 3:**
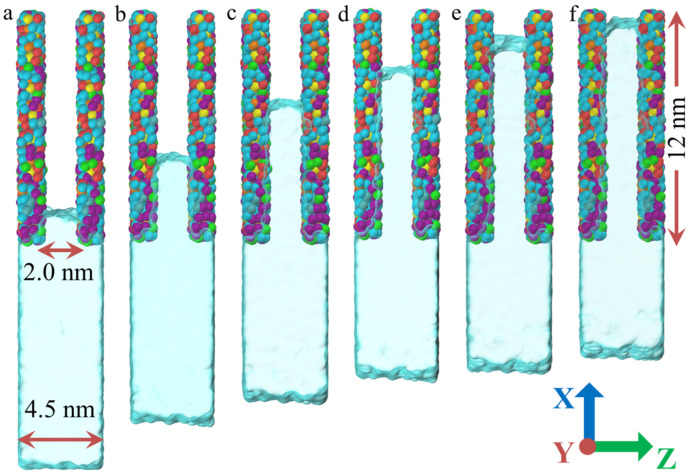
**Snapshot of the model at the maximum penetration depth of water molecules.** (**a**) 1.03 MPa. (**b**) 2.05 MPa. (**c**) 3.08 MPa. (**d**) 4.10 MPa. (**e**) 5.12 MPa. (**f**) 6.16 MPa.

As shown in Figure 2a, water pressure has a significant effect on the water density. In the initial stage of permeation, the water molecules do not immediately enter the PU nanochannels, as shown in Figure 2b. Instead, they are compressed under the influence of the water pressure, resulting in a gradual increase in density. Moreover, higher water pressure leads to a more pronounced increase in water density. As the permeation time increases, the energy of the water molecule system gradually rises, eventually breaking through the energy barrier and penetrating the channel. At this stage, the water density begins to decrease, and the higher the water pressure, the faster the rate of density reduction. However, at a pressure of 1.03 MPa, the water density remains nearly constant, indicating that the water molecules can maintain their original structural stability at this pressure. It is worth noting that once the permeation of the water molecules stabilizes, the average water density also reaches a stable value. As the water pressure increases, the stable water density also increases, surpassing that of bulk water. This is primarily because the water density at the entrance of the channel remains significantly higher than in other regions, as shown in Figure 2c. As permeation time increases, the number of water molecules entering the PU nanochannels gradually increases, as shown in Figure 2b. This process is closely related to water pressure, with higher water pressure resulting in a faster permeation of the water molecules (Figure 2d). It is also important to note that the penetration depth does not continue to increase indefinitely over time but stabilizes after reaching a certain threshold. Figure 3 shows snapshots of the water molecule models stabilized in PU nanochannels at various water pressures. It can be observed that the water molecules within the PU nanochannels form a convex liquid surface at the front, which is consistent with the hydrophobic properties of PU. Additionally, Figure 3 shows that the maximum permeation depth of the water molecules increases with water pressure, but the relationship is not linear. As the permeation pressure increases, the rate of increase in maximum permeation depth gradually decreases, as shown in Figure 2e. This suggests that the permeation behavior of the water molecules eventually reaches a saturation point, resulting in a decrease in permeation efficiency.

Figure 2f presents the velocity cloud map of the water molecules during permeation at a water pressure of 6.06 MPa. It can be observed that the velocity of the water molecules exhibits multi-stage characteristics. Before entering the PU nanochannels, the velocity of the water molecules outside the channels is relatively high due to the applied water pressure. However, due to the repulsive interaction between the PU and water molecules, their velocity gradually decreases as they approach the channel entrance. As the water molecules enter the PU nanochannels, the velocity of the water molecules both inside and outside the channels decreases significantly. However, the reduction in velocity is smaller inside the channels than outside. As permeation progresses, the velocity of the water molecules inside the channel increases again, leading to a second velocity peak (Figure 2g). This secondary acceleration in permeation is influenced by water pressure. The secondary acceleration of water molecule permeation occurs only when the water pressure exceeds 3.08 MPa, with higher pressures resulting in a more pronounced acceleration. Due to the hydrophobicity of the PU nanochannel surface, a significant difference in the velocity distribution of the water molecules is observed along the *Z*-axis, both inside and outside the channels, as shown in Figure 2h,i. The velocity distribution of the water molecules outside the channel is relatively uniform, with no noticeable stratification. However, within the channel, a distinct velocity stratification of the water molecules is observed along the *Z*-axis, with molecules farther from the channel surface exhibiting higher velocities. This is primarily attributed to the strong interaction between the water molecules near the channel wall and the PU molecules, which significantly limits the movement of the water molecules. An increase in water pressure exacerbates the stratification of water molecule velocity, with the velocity increase becoming more pronounced as the molecules move farther from the channel wall, especially at the center, where the increase is most significant. This is because the increase in water pressure imparts greater kinetic energy to the water molecules. The farther the water molecules are from the channel wall, the less they are restricted by wall interactions, allowing them to respond more effectively to external pressure and convert kinetic energy into velocity more easily. Meanwhile, as water pressure increases, more water molecules are pushed into the channels, exacerbating the velocity difference between the water molecules inside and outside the channels, which leads to a greater non-uniformity in the kinetic energy distribution within the channels.

### 3.2. The Effect of Channel Width

**Figure 4:**
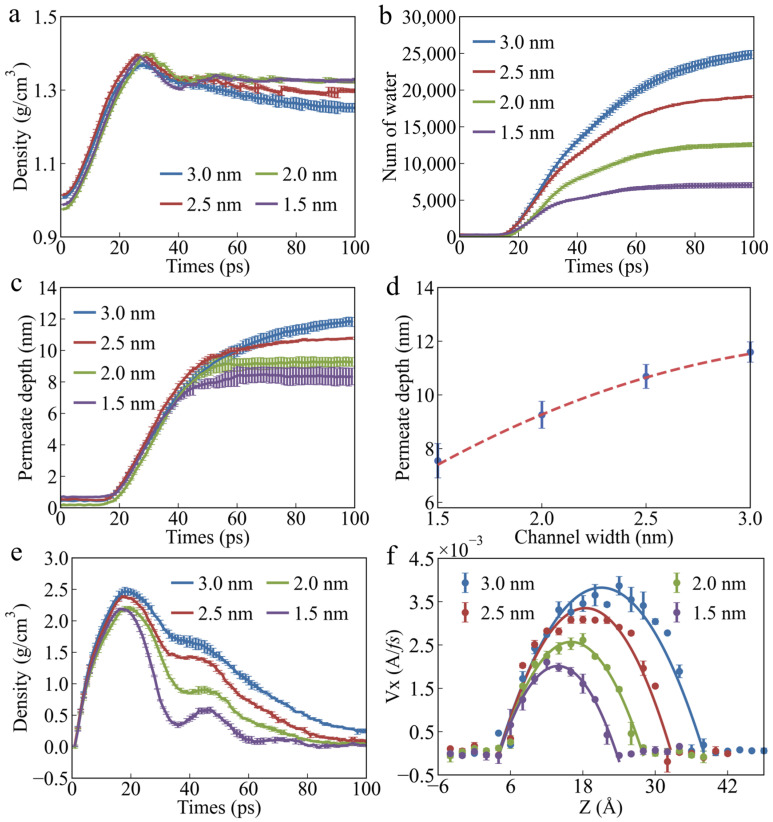
**Effect of channel width on the permeability characteristics of water molecules.** (**a**) Average water density as a function of permeation time. (**b**) Number of water molecules permeating PU nanochannels as a function of permeation time. (**c**) Permeation depth as a function of permeation time. (**d**) Maximum permeation depth as a function of channel width. (**e**) Average velocity of water molecules as a function of permeation time. (**f**) Distribution of the velocity of water molecules along the *Z*-axis inside nanochannels.

**Figure 5:**
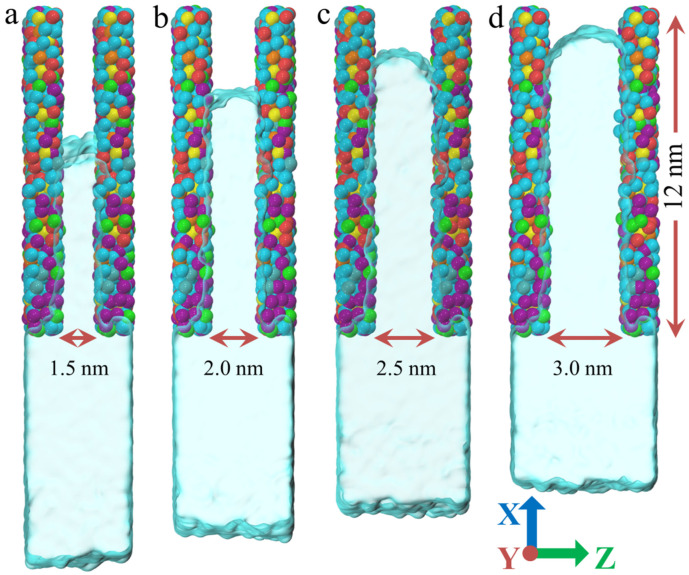
**Snapshot of the model at the maximum penetration depth of water molecules.** (**a**) 1.5 nm. (**b**) 2.0 nm. (**c**) 2.5 nm. (**d**) 3.0 nm.

Figure 4a illustrates the relationship between water density and permeation time during the permeation. It can be observed that in all PU nanochannel models, regardless of width, the water molecules initially experience a phase of increasing density. At this stage, the effect of channel width on water density is relatively small. This suggests that nanochannels of all widths exhibit energy barriers. In the initial stage of permeation, the water molecules are unable break through the energy barriers and are compressed by the water pressure. Subsequently, as the energy of the water molecules increases, they gradually break through the potential energy barrier and permeate the nanochannels. At this point, the water density begins to decrease and eventually reaches a stable state. Interestingly, the wider the channel, the lower the average water density in the stable state. The first reason is that an increase in channel width provides more space for the water molecules, allowing more water molecules at the entrance to penetrate the nanochannel. The second reason is that the larger space weakens the interactions between the water molecules, leading to a sparser distribution. The relationships between the number of water molecules permeating the nanochannels and permeation time, as well as the relationship between permeation depth and permeation time, are shown in Figure 4b and Figure 4c, respectively. It can be observed that the time required for the water molecules to break through the energy barrier in all models is approximately 20 ps, and this is not influenced by the channel width. After breaking through the energy barrier, the enhanced effect of increased nanochannel width on permeation begins to emerge. As the channel width increases, the permeation rate of the water molecules gradually increases. Figure 5 presents snapshots of the model with varying channel widths at the maximum penetration depth of the water molecules. It can be observed that the maximum penetration depth of the water molecules increases with the channel width, but this increase is not nonlinear. Instead, as the channel width increases, the growth rate of the maximum penetration depth gradually slows, as shown in Figure 4d. This is similar to the trend observed in the effect of water pressure on penetration depth. This occurs because, as the penetration depth of the water molecules increases, the proportion of the water molecules affected by the channel wall gradually decreases, weakening the effect of the increased channel width on penetration depth.

In PU nanochannels of all widths, the velocity changes of the water molecules exhibit distinct multi-stage characteristics, as shown in Figure 4e. The larger the channel width, the higher the first peak in water molecule velocity and the slower the rate of decrease in velocity after the water molecules break through the energy barrier. As the channel width increases, the flow rate of the water molecules in the nanochannel becomes faster, as shown in Figure 4f. The channel width significantly influences the secondary acceleration of water molecule permeation. With the increase in channel width, the secondary acceleration of water molecule permeation gradually decreases. A second velocity peak was observed for the water molecules in nanochannels with widths of 1.5 nm, 2.0 nm, and 2.5 nm. No secondary velocity peak was observed in the nanochannel with a width of 3.0 nm. This is because the increased width of the nanochannels provides more space for the distribution of the water molecules, reducing collisions and interactions between them, thereby lowering the frequency of momentum exchange. As a result, the overall energy transfer becomes smoother, which weakens the amplitude of secondary acceleration. Additionally, in channels of all widths, the velocity of the water molecules shows distinct layering characteristics along the *Z*-axis, as shown in Figure 4f. The farther the water molecules are from the channel surface, the faster the movement. As the channel width increases, the velocity of the water molecules farther from the channel wall increases even more significantly. As the distance from the channel wall increases, the water molecules gradually move out of the range of the wall effect, leading to reduced resistance and greater freedom. Therefore, interactions between the water molecules become dominant, enabling higher velocities through momentum transfer.

### 3.3. Motion and Structural Characteristics of Water Molecules in Confined Spaces

In this part, the MSD is used in this part to evaluate the mobility of atoms and is calculated using the following equations [52].

(7) $$MSD\left(t\right)=\frac{1}{N}\sum_{i=1}^{N}{\left[r_{i}\left(t+\Delta t\right)-r_{i}\left(t\right)\right]}^{2}$$

where *r_i_*(*t*) represents the position of atom *i* at time *t*, *N* represents the total number of atoms in the system, and Δ*t* represents the time step.

**Figure 6:**
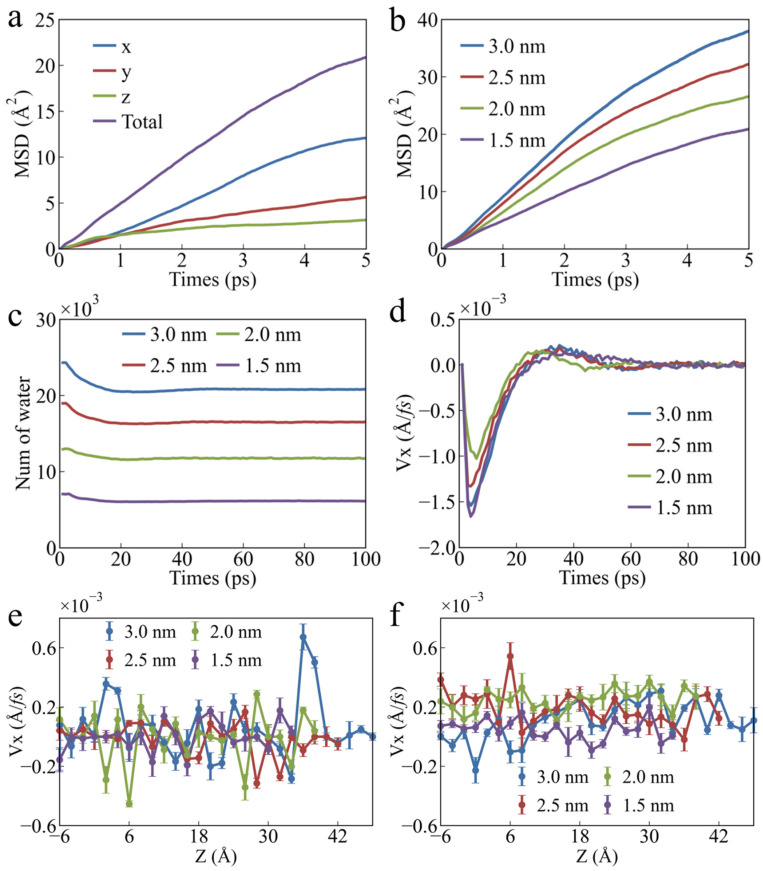
**Motion characteristics of water molecules inside and outside the nanochannel.** MSD of water molecules (**a**) inside nanochannel and (**b**) outside nanochannel. (**c**) Number of water molecules in nanochannel as a function of relaxation time after removing water pressure. (**d**) Velocity of water molecules in nanochannel as a function of relaxation time after removing water pressure. Distribution of velocity of water molecules along the *Z*-Axis (**e**) inside nanochannel and (**f**) outside nanochannel after removal of water pressure and stabilization.

Figure 6a shows the relationship between the MSD of the water molecules along the *X*-axis, *Y*-axis, and *Z*-axis within the nanochannel and the relaxation time. It can be observed that the water molecules exhibit the lowest mobility in the direction perpendicular to the channel wall, primarily due to the confining effect of the channel wall. Compared to the constraints imposed by H-bonds between the water molecules, the channel wall exerts a more significant restriction on their movement at the nanoscale. As the width of the nanochannel increases, the inhibitory effect of the channel wall on the water molecule movement decreases, leading to a gradual enhancement of the diffusion capacity of the water molecules, as shown in Figure 6b. In narrower channels, the confinement effect between the water molecules becomes more pronounced, leading to an increase in both the viscosity and the frequency of collisions with the channel walls, thereby restricting the diffusion capacity of the water molecules. Figure 6c illustrates the relationship between the number of water molecules in PU nanochannels and the relaxation time following the release of water pressure. A small portion of the water molecules flow out of the nanochannel, indicating that the water pressure inside the channel is higher than the external pressure. Consequently, the average velocity of the water molecules first increases and then decreases along the reverse permeation direction, eventually approaching zero and stabilizing, as shown in Figure 6d. After the removal of water pressure, the velocity of the water molecules inside the channel no longer exhibits stratification along the *Z*-axis. The velocity of the water molecules inside the channel is lower than that outside, which is consistent with the MSD calculation results, as shown in Figure 6b,e,f. This phenomenon is not only influenced by the restriction of water molecule movement by the channel wall but also closely linked to the structure of the water molecules in the confined space, including factors such as density distribution, H-bonds, and molecular orientation.

**Figure 7:**
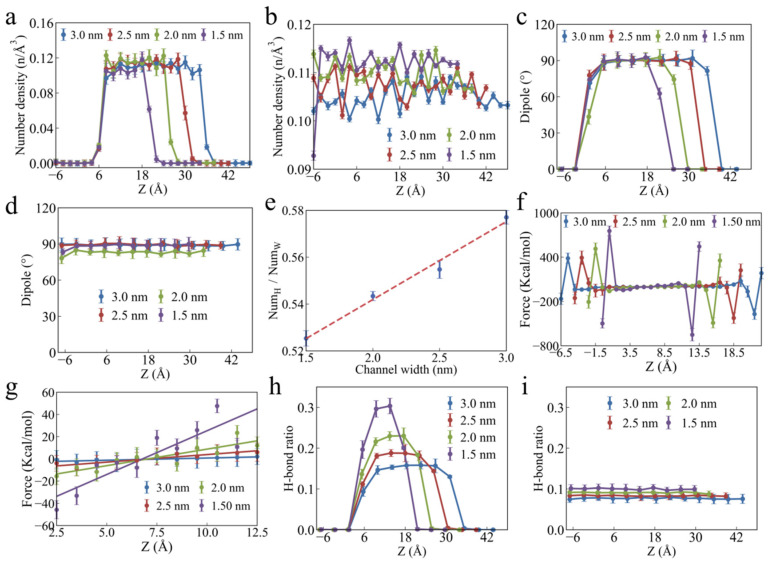
**Structure characteristics of water molecules inside and outside the nanochannel.** Distribution of number density of water molecules along the *Z*-axis (**a**) inside nanochannel and (**b**) outside the nanochannel. Distribution of dipole angle of water molecules along the *Z*-axis (**c**) inside nanochannel and (**d**) outside the nanochannel. (**e**) The relationship between the average number of H-bonds formed by water molecules in the channel and the channel width. Distribution of the force exerted by the channel walls on water molecules along the *z*-axis from (**f**) −6.5 nm to 22.5 nm and from (**g**) 2.5 nm to 12.5 nm. Distribution of H-bonds between water molecules along the *Z*-axis (**h**) inside nanochannel and (**i**) outside the nanochannel.

Figure 7a,b show the distribution of water molecule number density along the *Z*-axis inside and outside the nanochannel, respectively. A distinct hydrophobic layer can be observed near the nanochannel wall, and the number density of the water molecules in this hydrophobic layer is significantly lower than that in the middle region of the channel. The number density of the water molecules in the middle layer does not exhibit stratification, but the overall density is higher than that of the water molecules outside the nanochannel. Compared to the water molecules outside the nanochannel, the movement of the water molecules in the nanochannel is strictly confined by space. Additionally, the channel walls exert pressure on the molecules, causing them to compress inside the channel. This indicates that, in confined spaces, the water molecules accumulate more densely due to spatial constraints. As the channel height decreases, this stacking effect intensifies, and the viscous forces between the water molecules increase, resulting in reduced mobility. As shown in Figure 7c,d, changes in the dipole angle of the water molecules are primarily concentrated in the hydrophobic layer. The hydrophobicity of the PU channel wall influences the orientation of the water molecules within the hydrophobic layer, as well as enhances their interactions and reduces their mobility. Figure 7e shows the average number of H-bonds formed by each water molecule in the nanochannel. As the channel width decreases, the number of H-bonds formed by the water molecules also decreases. This is because, as the channel width decreases, the wall forces acting on the water molecules increase, significantly disrupting their interactions (Figure 7f). Additionally, the magnitude of the wall force acting on the water molecules is related to the distance from the molecule to the wall. The closer the water molecule is to the channel wall, the greater the wall force exerted on it (Figure 7g). The difference in wall force results in a layered structure of H-bonds between the water molecules in the nanochannel, with more H-bonds forming in areas farther from the wall (Figure 7h). In contrast, the H-bonds between the water molecules outside the nanochannel did not exhibit significant stratification (Figure 7i). The wall effect and confinement effect of the PU nanochannels jointly influence the structure and dynamic behavior of the water molecules, with a particularly pronounced impact in channels of smaller width.

## 4. Conclusions

In this study, a model combining a PU nanochannel and water molecules was constructed, and MD simulations were used to investigate the permeation behavior of water molecules in PU nanochannels under high water pressure. The microstructure of the water molecules within the PU nanochannels and the effects of water pressure and channel width on water molecule permeation behavior were analyzed. Due to the energy barriers in the nanochannels, the water molecules did not penetrate the PU nanochannels during the initial stage of the applied water pressure. Instead, they were compressed, resulting in an increase in the water density. Subsequently, the water molecules overcame the energy barrier and gradually penetrated the nanochannels, resulting in a gradual decrease in the water molecule density until it stabilized. When the water molecule density stabilized, it indicated that the maximum permeation depth had been reached. The greater the water pressure and nanochannel width, the higher the permeation velocity and the deeper the maximum penetration of the water molecules. The velocity of the water molecules during permeation exhibited multi-stage characteristics, including an initial acceleration followed by a secondary acceleration after entering the channel. The lower the water pressure and the wider the nanochannel, the less pronounced the secondary acceleration phenomenon became. Even when the water pressure was below 3.08 MPa and the nanochannel width reached 3 nm, the secondary acceleration phenomenon did not occur. The velocity of the water molecules within the channel exhibited clear stratification, with higher velocity occurring at greater distances from the nanochannel wall. An increase in water pressure and nanochannel width intensified the stratification of water molecule velocity. Due to the restrictive effect of the channel wall, the water molecules exhibited the lowest mobility in the direction perpendicular to the wall. This restrictive effect became more pronounced as the nanochannel width decreased. A hydrophobic layer approximately 0.5 nm thick formed near the wall, while the remaining area constituted the middle layer. The water density in the hydrophobic layer was significantly lower than that outside the nanochannel, while the density in the middle layer was slightly higher than that outside. Due to the effect of the channel wall, the angle between the dipole vector of the water molecules in the hydrophobic layer and the wall decreased, while the orientation of the water molecules in the middle layer remained unaffected. However, compared to the hydrophobic layer, the water molecules in the middle layer formed more H-bonds, with a significantly higher number than those formed between the water molecules outside the channel. Additionally, the H-bonds formed between the water molecules within the channel exhibited stratification, which was influenced by the variation in the wall force acting on the water molecules. The farther the distance from the channel wall, the greater the number of hydrogen bonds formed between the water molecules. Reducing the channel width enhanced the stratification of the hydrogen bonds within the channel.

## Data Availability

The relevant data generated and analyzed in the current study are available from the corresponding author upon reasonable request.
